# Chirality of nanophotonic waveguide with embedded quantum emitter for unidirectional spin transfer

**DOI:** 10.1038/ncomms11183

**Published:** 2016-03-31

**Authors:** R. J. Coles, D. M. Price, J. E. Dixon, B. Royall, E. Clarke, P. Kok, M. S. Skolnick, A. M. Fox, M. N. Makhonin

**Affiliations:** 1 Department of Physics and Astronomy, University of Sheffield, Hicks Building, Sheffield S3 7RH, UK; 2 EPSRC National Centre for III-V Technologies, Department of Electronic and Electrical Engineering, University of Sheffield, Sheffield S1 3JD, UK

## Abstract

Scalable quantum technologies may be achieved by faithful conversion between matter qubits and photonic qubits in integrated circuit geometries. Within this context, quantum dots possess well-defined spin states (matter qubits), which couple efficiently to photons. By embedding them in nanophotonic waveguides, they provide a promising platform for quantum technology implementations. In this paper, we demonstrate that the naturally occurring electromagnetic field chirality that arises in nanobeam waveguides leads to unidirectional photon emission from quantum dot spin states, with resultant in-plane transfer of matter-qubit information. The chiral behaviour occurs despite the non-chiral geometry and material of the waveguides. Using dot registration techniques, we achieve a quantum emitter deterministically positioned at a chiral point and realize spin-path conversion by design. We further show that the chiral phenomena are much more tolerant to dot position than in standard photonic crystal waveguides, exhibit spin-path readout up to 95±5% and have potential to serve as the basis of spin-logic and network implementations.

## Abstract (web summary)

Scalable quantum technologies require efficient conversion between qubits stored in solid-state systems and flying photonic qubits. Here, the authors demonstrate that the electromagnetic field chirality of a photonic waveguide leads to unidirectional emission from an embedded quantum dot emitter.

## Graphical abstract

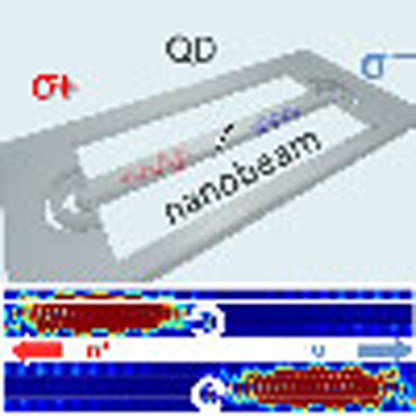

Quantum information processing promises a dramatically increased performance in computing, secure communications and simulations of quantum systems. In addition, a network of quantum nodes enables distributed quantum computing^1^, and may facilitate the quantum internet^2^. Such a distributed architecture requires the conversion between matter qubits for local quantum memories and photonic qubits for quantum communication between nodes^3^,^4^,^5^. Furthermore, any scalable quantum technology must solve the miniaturization and fabrication problem, which likely demands that the quantum nodes must be implemented on integrated circuits and waveguides^6^. Recent developments of passive components^7^ with embedded quantum dots (QDs) using advanced semiconductor technologies and enhanced-coherence using resonant techniques on-chip^8^ contribute to potential solutions. The spin of an electron or hole in a QD is a promising candidate to serve as the matter-qubit in a quantum network, but its implementation requires an efficient on-chip spin–photon interface.

The coupling of spin to the direction of photon emission in nanophotonic waveguides provides a potential solution. The subject of unidirectional light propagation in nanophotonic structures under circularly polarized laser excitation has been advancing rapidly since the first reports in 2013 for surface plasmons and atomic dipoles^9^,^10^. The chiral effect is understood to arise from the longitudinal component of evanescent fields in nanophotonic structures, which enables circularly polarized states to propagate with the field rotating within the longitudinal plane defined by the geometry of the waveguide^9^,^10^,^11^,^12^,^13^,^14^,^15^,^16^. An important step forward was made very recently in which directional emission was demonstrated for a quantum-dot emitter embedded within a specially engineered glide-plane waveguide^14^, confirming theoretical predictions for chiral emission in photonic crystal waveguides (PhC WGs)^14^,^15^.

In this work, we demonstrate the internal intrinsic chirality of the electromagnetic field in a system with no specially engineered chirality using the QD as an internal probe. We demonstrate the importance of the position of the quantum emitter, in such a way that spin-dependent directional emission is achieved. We achieve efficient coupling of QD exciton spin to the direction of photon emission in nanophotonic waveguides. This chiral behaviour occurs even though the waveguide geometry is completely symmetric, and the dielectric material is non-chiral. We use numerical simulations to demonstrate that the chiral emission originates from the chirality of the electromagnetic field inside the waveguide and exploit it for efficient coupling of the QD emitter by lateral positioning within the waveguide. The dots located at the centre of the waveguide exhibit non-chiral emission, but those displaced from the centre show a varying degree of chiral emission with distance from the centre. The maximal spin-dependent directionality occurs for QDs located at chiral points shifted from the waveguide centre by 32% of the waveguide width. We first confirm this hypothesis by measuring the variation of the spin-dependent directionality of a large number of dots, with good agreement found between the experimental data and the predictions of simulations for randomly positioned emitters within the waveguide. We compare results for nanobeam and PhC WGs, and find that the simpler nanobeam structure provides substantially better spin-dependent directionality because of the lower sensitivity of the chirality to the exact dot position. We then demonstrate designable spin-path coupling by using enhanced registration techniques^17^,^18^ (see the Methods for details) to achieve highly directional emission for a QD deterministically positioned at a chiral point. We conclude that directionality factors approaching 100% (95±5%) are achievable for the exciton spin of an embedded dot to a single photon state propagating in a nanophotonic waveguide. We also confirm the expected bi-directional emission for a dot deterministically positioned at the centre of the waveguide. We further note that our results provide a natural explanation for the bi-directional emission reported but not explained in cross-waveguide structures^19^. Taken together, the results establish a route towards the transfer and entanglement of the spin state of an emitter to a photonic qubit in an integrated network.

## Results

### Directional emission in dielectric nanobeam waveguides

**Figure 1:**
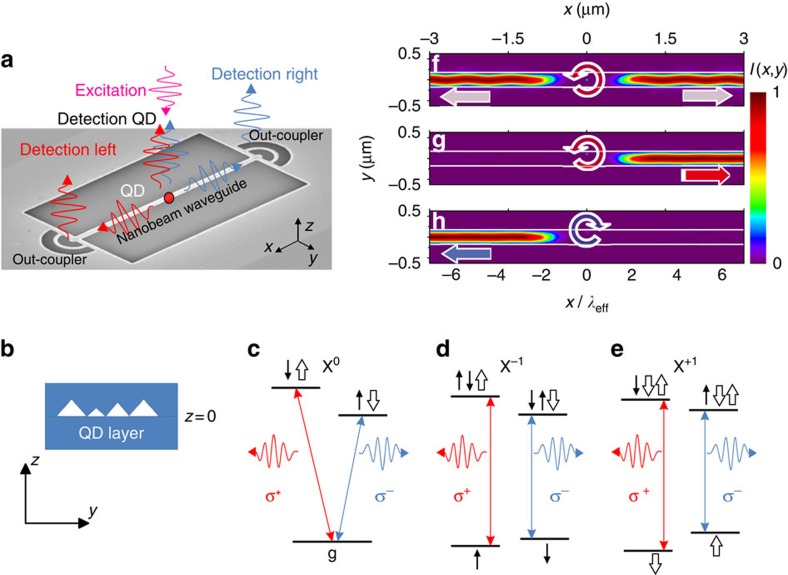
Directional emission in dielectric nanobeam waveguides. (**a**) Schematic representation of the experiments on directional emission from quantum dots embedded inside a single-mode nanobeam waveguide (NWG) with out-couplers for photon collection. Red (blue) arrows correspond to photons originating from right (left) circularly polarized dipoles in the quantum dot. Scheme labels are plotted on top of an SEM image of the real device. (**b**) A schematic of *yz* cross-section of the NWG with the QD layer at *z*=0. Level diagrams and optical transitions for (**c**) neutral (X^0^) and (**d**,**e**) charged (X^−1^) and (X^+1^) exciton QD states in **B**≠0. (**f**) Time averaged intensity distribution of the emission *I*(*x,y*) (colour scale) in the *xy*-plane at *z*=0 from circularly polarized σ^+^ dipole at the centre of the NWG (*y*=0). (**g**,**h**) Emission for a circularly polarized dipole at the chiral point, displaced from the centre of the waveguide by 32% of the waveguide width: σ^+^ dipole; σ^−^ dipole. The undulation arises from periodic coupling with time of the rotating circular dipole at the non-chiral point. The intensity distributions in (**f**–**h**) are calculated after the dipole source is switched off. The *x* axis is normalized to the NWG mode effective wavelength *λ*_eff_. The white horizontal lines show the nanostructure boundaries. Fully animated versions of the simulations may be found in the Supplementary Notes 1–6.

The system we investigate is a QD in a nanophotonic waveguide. The dot serves as an integrated quantum emitter with addressable spin-exciton eigenstates, generating spin- and position-dependent directional emission of single photons in the waveguide by exciton recombination. Nanophotonic structures support both longitudinal and transverse field components (*E*_*x*_ and *E*_*y*_) and, as we show, permit the transfer of in-plane circular polarization (*E*_*x*_±*iE*_*y*_; see Fig. 1a,b for definition of axes). Despite its non-chiral geometry and material, the waveguide exhibits a chiral electromagnetic field mode. As a result, a well-positioned QD will emit in-plane circularly polarized single photons unidirectionally, with a high correlation between the spin state of the QD and the which-path information of the photon.

A schematic of the experimental geometry is given in Fig. 1a. A cross-section of the device including the layer of QDs is shown schematically in Fig. 1b. The left- and right-circularly polarized photons arising from recombination of up and down exciton spin states from a QD embedded within a suspended nanobeam waveguide (NWG) are coupled to NWG modes, and then diffracted by out-coupler gratings at opposite ends towards external detectors. As shown in Fig. 1c–e, for all three charge states of the dots, exciton recombination leads to circularly polarized photon emission. In a non-chiral structure, the emission probability for a circularly polarized dipole is identical in both directions, and this behaviour is indeed observed when the dot is located at the centre of the waveguide (see Fig. 1f obtained from finite difference time domain (FDTD) simulations, see the Methods). By contrast, when the dot is displaced from the centre of the waveguide, the emission direction depends on the spin, with σ^+^ photons emitted in one direction and σ^−^ in the other. The unidirectional emission is shown in Fig. 1g,h (the results of FDTD simulations), and occurs through the coupling of the σ^+^/σ^−^ dipole emitters to direction-dependent modes at the chiral points of the NWG.

### Electric field distributions in NWG

**Figure 2:**
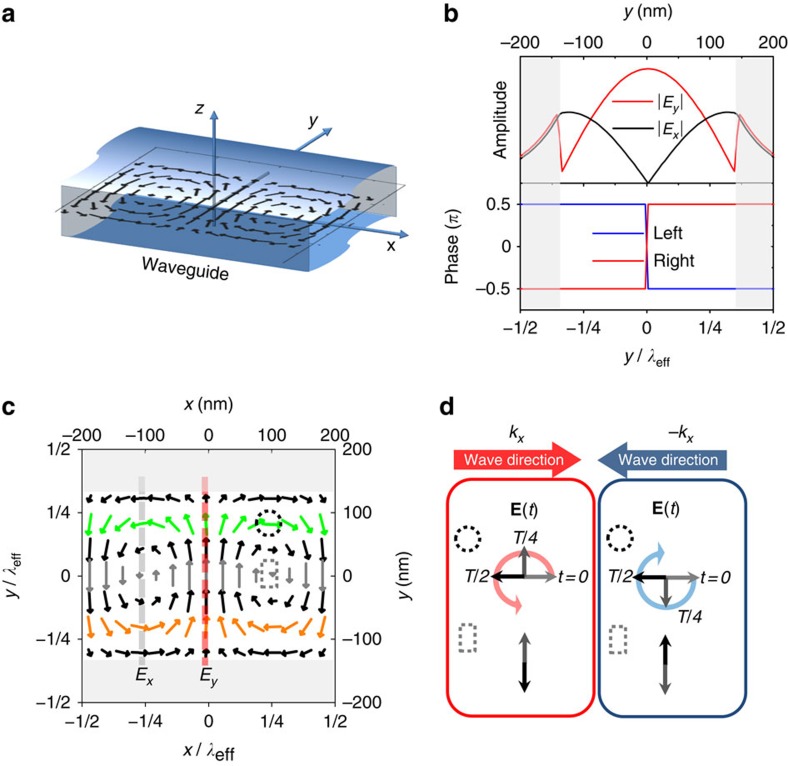
Electric field distributions in nanobeam waveguide. (**a**) Schematic of an infinite length NWG showing the orientation of the axes and the field distribution in the *xy*-plane at *z*=0, as defined by the central layer of the waveguide containing QDs. (**b**) Electric field amplitudes (top) for the *x* and *y* components and their relative phase (bottom) for left/right propagating modes. (**c**) Simulated distribution of the electric field vector for the first fundamental mode at a fixed moment in time. Arrows show the direction of the field and the length of the magnitude. Colour is used as a guide to the eye: green/orange for opposite-helicity chiral points, and grey for central points with linear polarization. The left/bottom scales are normalized to the NWG mode effective wavelength *λ*_eff_. (**d**) Electric field time evolution in space from **c** for propagating modes at chiral (circular dotted area) and non-chiral (rectangular dotted area) points in the NWG. The red and blue arrows show the *k*_*x*_ and *–k*_*x*_ mode propagation direction, respectively. The rotation of the electric field vectors exhibiting circular right/left polarization is shown by red/blue circular arrows. The grey areas in **b** and **c** correspond to air cladding regions at the NWG edges.

The origin of the unidirectional emission can be understood by consideration of the electromagnetic field distribution within the laterally confined nanophotonic geometry. Figure 2a shows a schematic representation of an unterminated NWG with the field distribution in the *xy*-plane at *z*=0 (position of the QDs layer) calculated by solving Maxwell's equations (Supplementary Note 1). The amplitudes of the *E*_*y*_ and *E*_*x*_ field components, together with their relative phases, are shown in Fig. 2b. The |*E*_*y*_| component has a maximum at the centre of the NWG, whereas |*E*_*x*_| has two maxima closer to the edges (Supplementary Note 2). The relative phase between *E*_*x*_ and *E*_*y*_ is constant at either ±*π*/2 and changes sign when crossing *y=*0. It is this asymmetry in the phase that enables chiral behaviour: the field is right- or left-circularly polarized (that is, σ^±^ corresponding to *E*_*x*_±*iE*_*y*_ fields) at points where |*E*_*y*_|=|*E*_*x*_| and the phase is ±*π*/2. This is shown more clearly in Fig. 2c, where the electric field is plotted vectorially at an instant in time. The chiral points are positions where the electric field rotates in time during propagation of the waveguide mode. The rotation direction depends on the propagation direction as indicated in Fig. 2d, so that σ^±^ emitters couple preferentially to modes propagating in opposite directions. By contrast, the point at the centre of the waveguide (*y*=0) has no longitudinal component (*E*_*x*_=0) and is therefore linearly polarized (Fig. 2c,d). Translation along *y* from the centre to the chiral points thus transforms the field polarization from linear, through elliptical, to circular.

### Spin readout in a NWG and PhC WG

**Figure 3:**
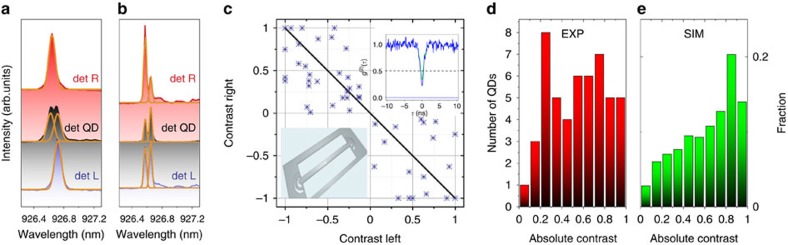
Experimental demonstration of spin readout in nanobeam waveguides. (**a**) Photoluminescence spectra of QDs with high contrast in NWGs. (**b**) Experimental photoluminescence spectra of QDs with low contrast in NWGs. Fitted peaks in **a** and **b** are shown with orange curves. (**c**) Readout contrast *C=*(*I*_σ+_*–I*_σ−_)/(*I*_σ+_*+I*_σ−_) for left and right out-couplers for randomly distributed self-assembled QDs in NWGs (50 QDs). The points correspond to experimental data, and the black diagonal line shows the expected distribution for ideal circularly polarized dipoles. (**d**) Statistical distributions of the absolute average contrasts *C*=(|*C*_LEFT_|+|*C*_RIGHT_|)/2 of QD spin readout taken from the experimental data in **c** for NWGs. All experimental data are recorded at *B*_*Z*_*=*1 T. (**e**) Simulated statistical distributions of absolute contrasts calculated from the coupling of circularly polarized dipoles distributed across the WGs in the *xy*-plane (*z*=0) taking into account the electric field distributions (Supplementary Note 3) due to the out-couplers for NWG. The top inset in **c** shows a typical QD autocorrelation function *g*^(2)^(*τ*) (blue curve with background subtraction *g*^(2)^(0)=0.22, green without *g*^(2)^(0)=0.34). The inset in **c** shows schematic of the NWG.

**Figure 4:**
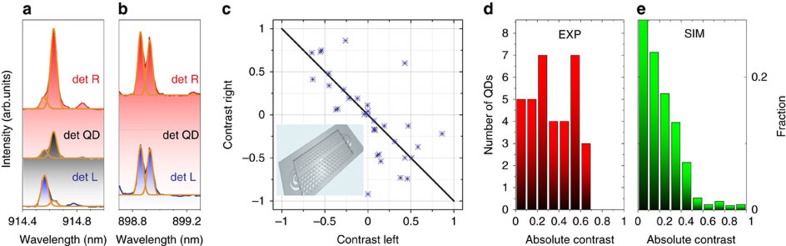
Experimental demonstration of spin readout in photonic crystal waveguides. (**a**) Photoluminescence spectra of QDs with high contrast in PhC WGs. (**b**) Experimental photoluminescence spectra of QDs with low contrast in PhC WGs. Fitted peaks in **a** and **b** are shown with orange curves. (**c**) Readout contrast *C=*(*I*_σ+_*−I*_σ−_)/(*I*_σ+_*+I*_σ−_) for left and right out-couplers for randomly distributed self-assembled QDs in PhC WGs (35 QDs). The points correspond to experimental data, and the black diagonal line shows the expected distribution for ideal circularly polarized dipoles. (**d**) Statistical distributions of the absolute average contrasts *C*=(|*C*_LEFT_|+|*C*_RIGHT_|)/2 of QD spin readout taken from the experimental data in **c** for PhC WGs. All experimental data are recorded at *B*_*Z*_*=*1 T. (**e**) Simulated statistical distributions of absolute contrasts calculated from the coupling of circularly polarized dipoles distributed across the WGs in the *xy*-plane (*z*=0) taking into account the electric field distributions (Supplementary Note 4) due to the out-couplers for PhC WGs. The inset in **c** shows schematic of the PhC WGs.

To demonstrate the chiral effects experimentally, we use a single-mode NWG with randomly distributed self-assembled QDs, positioned by growth in the *xy*-plane at *z*=0. The guided photoluminescence (PL) of single QDs is detected from out-couplers at opposite ends of the NWG (Fig. 1a). The inset in Fig. 3c shows a typical autocorrelation function *g*^(2)^(*τ*) confirming the single-photon nature of the QD emission. The sample was placed in an out-of-*xy*-plane magnetic field *B*_*z*_ to quantize the QD spins into *S*_*z*_*=*±1 states (spin up/down). This enables identification of exciton Zeeman spin components from the energy of the circularly polarized (σ^+^/σ^−^) photons emitted: *hv*_x_(*S*_z_)=*hv*_0_+*μ*_B_*g*_x_*B*_z_S_z_, where *g*_X_ is an excitonic g-factor that varies from dot to dot with a typical value of ∼1.2. *B*_*Z*_*=*1 T for the data in Figs 3 and 4. Self-assembled growth leads to random positions of QDs, enabling the observation of emission from both chiral and non-chiral areas of the waveguide. Figure 3 contrasts the behaviour for two dots selected from the random distribution, with one showing chiral behaviour (Fig. 3a), and the other not (Fig. 3b). For the QD in the chiral point in Fig. 3a, both Zeeman-split components are seen when detecting directly from the dot (‘det QD'), but only one component is observed for detection from the left and right out-couplers (‘det L' and ‘det R'). The two Zeeman components emit in opposite directions, implying spin-dependent directional emission. By contrast, non-directional emission is observed for the dot in centre of the waveguide (Fig. 3b), with both Zeeman components detected from both out-couplers. The degree of contrast in spin-readout (the directionality factor) is defined as:


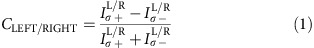


where the superscripts L and R refer to the left and right out-couplers. The results for 50 QDs are shown in Fig. 3c. The diagonal line across the figure is the expectation for a random distribution of dots in the *xy*-plane at *z*=0 whose circular dipoles couple to the confined electromagnetic fields. QDs at the linear point at the centre of the waveguide correspond to zero contrast, whereas dots at the chiral points give rise to contrasts of ±1. Agreement between the trends in contrast between experiment and simulation is seen (the experimental scatter around the diagonal is discussed below).

The data from Fig. 3c are plotted as a histogram of numbers of dots of absolute contrast *C=*(*|C*_LEFT_*|+|C*_RIGHT_|)/2 in Fig. 3d and compared with binned data of the FDTD simulations of contrast (see Supplementary Fig. 7 for details) for out-coupler-terminated waveguide in Fig. 3e. Details of the comparison between infinite and terminated waveguides are given in the Supplementary Notes 2 and 3. The out-coupler gratings introduce back-reflections that lead to modulation of the field distributions; they do not change the mechanisms responsible for unidirectionality, but result in reduction in the probability for dots with high (>90%) spin readout conversion by ∼52%. Qualitative agreement between experiment (Fig. 3d) and simulation (Fig. 3e) is observed, with an increasing number of QDs to high contrast clearly seen, and levelling-off around a contrast of 80%. It is also notable that QDs with extremely high directionality exceeding 90% are observed experimentally (Fig. 3c,d). These results support the findings in the simulations which show chiral areas (Supplementary Fig. 6d) of 14% of the waveguide where the contrast exceeds 90% (and 34% exceeding 80%).

The circular dipole unidirectional coupling efficiency, defined as the fraction of power emitted into a waveguide mode propagating in one direction, is calculated to be 68% for a dipole positioned at a chiral point of the NWG (simulations in the Methods). This is an increase of more than ∼2.8 compared with the coupling of a circular-dipole at the centre of the waveguide into one spatial direction. It is also ∼1.4 times greater than for a linear-dipole co-polarized with the field at the centre of the NWG (*y*=0)^8^. Both comparisons show the favourable degrees of coupling efficiency obtained for spin readout at the chiral points.

In order to compare to PhC WGs, we perform similar experiments and simulations for them. QD spectra with high/low contrasts are shown in Fig. 4a,b. The contrasts for the 35 QDs studied are shown in Fig. 4c. The overall trends in the behaviour are similar to those seen in Fig. 3c for the NWGs, but with the very marked difference that no QDs exhibit high directionality values above 70% (Fig. 4c,d). This is expected as the chiral areas (Supplementary Fig. 9d) inside the PhC WG, where the contrast exceeds 90%, only constitute ∼0.8% of the waveguide area (∼1.5% of area for >80% contrast) as shown in Supplementary Note 4. They are furthermore located in areas of low field intensity (Supplementary Fig. 9a), leading to poor dipole coupling. Both the high-fidelity chiral areas (∼20 times smaller than for the NWGs) and field intensities for the PhCs indicate the advantageous characteristics of the NWGs for spin readout, with their near continuous translational symmetry, relative to PhC WGs. This situation can likely be improved for PhC WGs by the use of glide plane techniques, as shown in ref. 14.

### QD registration and spin readout

**Figure 5:**
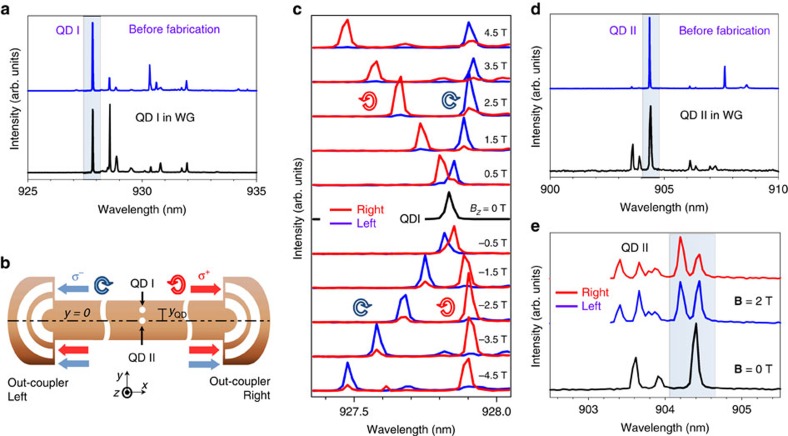
Quantum dot registration and spin readout. (**a**) Spectra of QD I before and after NWG fabrication. (**b**) Schematic of spin readout for registered QD I displaced vertically from the centre by 32% of the waveguide width (the Sample details in the Methods) and the absence of spin-readout for registered QD II located at the centre of the waveguide. (**c**) Magnetic field dependence of emission from the registered QD I collected from the left out-coupler (blue) and right out-coupler (red). (**d**) Spectra of QD II before and after NWG fabrication. (**e**) Spectra of QD II in magnetic field **B**=2 T collected from left and right out-couplers and spectrum of QD II at **B**=0 T for reference.

Having identified the location of chiral points theoretically, and observed high contrast unidirectional emission for randomly distributed dots, we now demonstrate control of the emission direction, using QD registration techniques^17^,^18^ (see the Methods for details). First, QD spectra were compared before and after the registration and NWG fabrication to confirm that the QD (we term this dot QD I) is successfully integrated inside the NWG (Fig. 5a). The waveguide is fabricated (see the Methods for details) such that QD I is displaced from the centre of the waveguide at the chiral point, with *y*_QD_ ∼30% of the waveguide width (see Fig. 5b schematic for details). For this position, we expect the σ^+^ dipole to couple to the right propagating mode and the σ^−^ dipole to the left (Fig. 5b). The spectra in magnetic field reveal high directionality (|*C*_LEFT_|=92±3%, |*C*_RIGHT_|=80±3%) of emission with absolute contrast independent of magnetic field to within 10%. Furthermore, the expected signs of the contrast correlate with the QD I position, with the σ^+^ line appearing at the right out-coupler with high energy when the field is positive, whereas the σ^−^ line is collected from the left out-coupler. Moreover, when the direction of the magnetic field is changed, the order of the lines is reversed, but the dipole coupling direction remains the same (Fig. 5c). Thus, we not only achieve control of spin-directionality by registration but also the reversal of the emission direction at a given energy by magnetic field control^14^. We further demonstrate the effectiveness of the registration techniques by fabricating a control sample with a QD (QD II) positioned at the centre of the waveguide (see Fig. 5b schematic for details). Spectra before and after NWG fabrication are compared to prove the integration of the QD within the waveguide (Fig. 5d). Low spin-readout contrast of (|*C*_LEFT_| ∼3±6%, |*C*_RIGHT_|=24±4%, Fig. 5e) is found, as expected for a dot at the centre of the waveguide. We note that the approach with dot registration is not limited to one QD and can be scaled up to create more complex circuits with deterministically coupled QDs to realize spin–photon and spin–spin entanglement on chip.

## Discussion

As noted above, the experimental points in Figs 3c and 4c show scatter around the diagonal, compared with theoretical expectations. This behaviour is not fully understood. It may arise due to back reflections in the finite length waveguides, in combination with elliptically polarized QDs. Fine structure splitting^20^ of the QDs can be excluded as we observe no magnetic field dependence for asymmetrically coupled QDs. The circular polarization for the QDs may also be affected in photonic structures by the nanostructure and surface proximity^21^. Moreover, the finite size of the dot, which is known to cause a breakdown in the point dipole approximation^22^ used for our simulations in nanophotonic structures may also affect the contrast.

To conclude, we demonstrate experimentally and theoretically chiral effects in a simple nanophotonic system consisting of waveguides containing embedded quantum emitters. The unidirectional phenomena we report may be used for spin read out and to transfer spin information from localized emitters in waveguide geometries. Deterministic positioning of a QD at a chiral point of the waveguide is achieved, with accompanying spin readout, a possible route to scalability. Larger areas for chiral behaviour found in the NWGs because of their continuous translational symmetry together with simplified fabrication techniques and low loss may provide significant advantages over PhC WGs. The findings and techniques presented could contribute to the creation of spin-optical on-chip networks and processing devices based on nanophotonic waveguides.

## Methods

### Sample details

The sample was grown by molecular beam epitaxy on an undoped [100] GaAs substrate, and consisted of a 140-nm-thick GaAs membrane containing a single layer of self-assembled InGaAs QDs at the centre grown on a 1-μm sacrificial Al_*0.6*_Ga_*0.4*_As layer. The photonic structures were patterned by electron beam lithography (EBL) and the pattern transferred to the membrane using inductively coupled plasma etching. To release the membranes, the sacrificial AlGaAs layer was removed by selective wet etch using a buffered hydrogen fluoride solution. The suspended NWGs were 15 μm long, 280 nm wide and 140 nm height. An scanning electron microscopy (SEM) image of the suspended NWG is shown in Supplementary Fig. 1a. The W1 PhC WGs were fabricated with lattice constant *a*=254 nm and hole radius *r*=0.31 *a*. An SEM image of the PhC WG is presented in Supplementary Fig. 1b. Both waveguides were terminated with semi-circular *λ*/2*n* air-GaAs out-coupler gratings designed for optimum operation at *λ*=950 nm at the centre of the QD ensemble PL emission^23^.

### Experimental set-up

All measurements were performed in a helium bath cryostat at *T*=4.2 K within which a superconducting magnet provided magnetic field of up to 5 T normal to the sample plane. Ultra-stable positioning of the sample within the system is provided by X, Y, Z home-made piezo stages. The cryostat insert had optical access to the sample in a confocal scanning microscope arrangement^24^. The excitation and collection spots were below 1.5 μm in diameter and could be separately moved by more than 15 μm by scanning mirrors to obtain the exact geometry required for each experiment. Optical excitation was provided by an 808 nm diode laser coupled to an optical fibre. The collected PL signal was fibre coupled and spectral measurements were performed using a spectrometer with a LN2 cooled charged-coupled device (CCD). Using a second exit port on the spectrometer, spectrally filtered PL signals were sent to two avalanche photodiodes (APDs) for QD autocorrelation *g*^(2)^*(τ)* measurements using a single-photon counting module.

### QD registration method

Dot registration^17^,^18^ is carried out in a scanning micro-PL set-up with two collection paths, as illustrated in Supplementary Fig. 2. The dot registration process involves three stages: pre-registration markers are fabricated, individual QDs are registered and photonic structures are deterministically fabricated around a QD. Registration markers are patterned using EBL and a positive resist. The sample is developed in xylene leaving the wafer surface exposed in the desired pattern, as shown in Supplementary Fig. 3a. The inner double markers are used to register the relative position of a QD, whereas the outer angular markers are used to re-align the EBL during the final fabrication stage. A layer of 5 nm of titanium followed by 20 nm of gold are then evaporated onto the wafer. The sample is then placed in a solvent bath to remove the remaining layer of resist and any unwanted titanium and gold.

A 1-mm diameter cubic zirconia (ZrO_2_) Weierstrass solid immersion lens is placed directly above a registration grid, with an aberration free image size of ∼50 μm in diameter. A low QD density of ∼1.6·10^7^ cm^−2^ and a solid immersion lens-enhanced excitation spot size ∼350 nm enables individual QDs to be measured. The closed-loop three axes scanning piezo stage, with a resolution of 1 nm, is used to scan the objective lens, which simultaneously moves the excitation and collection spots. One collection path is spectrally filtered through a monochromator to isolate a single exciton line before it is measured with an APD. The second collection path is used to measure the reflected laser signal from the gold markers to provide reference points in the scan from which the QD position is measured. No additional spectral filtering is used on the second collection path before it is detected with an APD, although it is heavily attenuated using neutral density filters. Horizontal and vertical line scans are used to determine the relative QD position. The signals from both APDs were recorded during the scans as a function of the stage position (Supplementary Fig. 3b).

The spacing between the gold markers is designed to be 15 μm. The relative position of the QD is then determined by repeating the line scans 100 times to provide a statistical average (Supplementary Fig. 3c). A Gaussian curve is fitted to the distribution to determine the centre. The minimum standard deviation observed is *σ*<5 nm, which is exceeding the accuracy of the best position uncertainty previously achieved^17^,^18^. The position of the suspended NWGs to be fabricated is placed within the original EBL design file at a suitable location, such that the QD are in the centre or laterally displaced. To align the EBL writing field to the pattern previously fabricated, the electron beam is scanned over the outer (blue) registration markers. The reflected electron flux is recorded and used to determine the central position of each marker. The suspended nanobeam is then fabricated (SEM image in Supplementary Fig. 3d) as described above in the Sample details section.

### Calculations of the fields in the waveguides

FDTD simulations were performed using freely available software package^25^ and commercial-grade simulator^26^. The GaAs waveguide devices simulated had the dimensions described in the Sample details section above. Details of the simulations are available in Supplementary Notes 1–4.

## Additional information

**How to cite this article**: Coles, R. J. *et al.* Chirality of nanophotonic waveguide with embedded quantum emitter for unidirectional spin transfer. *Nat. Commun.* 7:11183 doi: 10.1038/ncomms11183 (2016).

## Supplementary Material

Supplementary Information Supplementary Figures 1-10, Supplementary Notes 1-4 and Supplementary References.

Supplementary Movie 1 Electric field intensity of the suspended nanobeam waveguide in the xyplane at z = 0 for a d+ (σ+ polarised) dipole source located at y = +90 nm. The area shown is x = 8 μm by y = 2 μm.

Supplementary Movie 2 Electric fields intensity of the suspended nanobeam waveguide in the xyplane at z = 0 for a d- (σ- polarised) dipole source located at y = +90 nm. The area shown is x = 8 μm by y = 2 μm.

Supplementary Movie 3 Electric fields intensity of the suspended nanobeam waveguide in the xyplane at z = 0 for a d+ (σ+ polarised) dipole source located at y = 0, the centre of the waveguide. The area shown is x = 8 μm by y = 2 μm.

Supplementary Movie 4 Electric fields intensity of the photonic crystal waveguide in the xy-plane at z = 0 for a d+ (σ+ polarised) dipole source located at a chiral-point within the waveguide. The area shown is x = 8 μm by y = 2 μm

Supplementary Movie 5 Electric fields intensity of the photonic crystal waveguide in the xy-plane at z = 0 for a d- (σ- polarised) dipole source located at a chiral-point within the waveguide. The area shown is x = 8 μm by y = 2 μm.

Supplementary Movie 6 Electric fields intensity of the photonic crystal waveguide in the xy-plane at z = 0 for a d+ (σ+ polarised) dipole source located at a linear-point at y = 0. The area shown is x = 8 μm by y = 2 μm.

## References

[b1] Barz S. *et al.* Demonstration of blind quantum computing. Science 335, 303–308 (2012). 22267806 10.1126/science.1214707

[b2] Kimble H. J. The quantum Internet. Nature 453, 1023–1030 (2008). 18563153 10.1038/nature07127

[b3] Kok P. & Lovett B. W. Introduction to Optical Quantum Information Processing Cambridge Univ. (2010).

[b4] DiVincenzo D. P. The physical implementation of quantum computation. Fortschr. Phys. 48, 771–783 (2000).

[b5] Barrett S. D. & Kok P. Efficient high-fidelity quantum computation using matter qubits and linear optics. Phys. Rev. A 71, 060310 (2005).

[b6] O'Brien J. L. Optical quantum computing. Science 318, 1567–1570 (2007). 18063781 10.1126/science.1142892

[b7] Prtljaga N. *et al.* Monolithic integration of a quantum emitter with a compact on-chip beam-splitter. Appl. Phys. Lett. 104, 231107 (2014).

[b8] Makhonin M. N. *et al.* Waveguide coupled resonance fluorescence from on-chip quantum emitter. Nano Lett. 14, 6997–7002 (2014). 25381734 10.1021/nl5032937

[b9] Rodriguez-Fortuño F. J. *et al.* Near-field interference for the unidirectional excitation of electromagnetic guided modes. Science 340, 328–330 (2013). 23599487 10.1126/science.1233739

[b10] Junge C., O'Shea D., Volz J. & Rauschenbeutel A. Strong coupling between single atoms and nontransversal photons. Phys. Rev. Lett. 110, 213604 (2013). 23745874 10.1103/PhysRevLett.110.213604

[b11] Mitsch R., Sayrin C., Albrecht B., Schneeweiss P. & Rauschenbeutel A. Quantum state-controlled directional spontaneous emission of photons into a nanophotonic waveguide. Nat. Commun. 5, 5713 (2014). 25502565 10.1038/ncomms6713 PMC4284658

[b12] Petersen J., Volz J. & Rauschenbeutel A. Chiral nanophotonic waveguide interface based on spin-orbit coupling of light. Science 346, 67–71 (2014). 25190718 10.1126/science.1257671

[b13] le Feber B., Rotenberg N. & Kuipers L. Nanophotonic control of circular dipole emission. Nat. Commun. 6, 6695 (2015). 25833305 10.1038/ncomms7695

[b14] Söllner I. *et al.* Deterministic photon–emitter coupling in chiral photonic circuits. Nat. Nano 10, 775–778 (2015). 10.1038/nnano.2015.159 26214251

[b15] Young A. B. *et al.* Polarization engineering in photonic crystal waveguides for spin-photon entanglers. Phys. Rev. Lett. 115, 153901 (2015). 26550722 10.1103/PhysRevLett.115.153901

[b16] Bliokh K. Y., Rodríguez-Fortuño F. J., Nori F. & Zayats A. V. Spin–orbit interactions of light. Nat. Photon 9, 796–808 (2015).

[b17] Thon S. M. *et al.* Strong coupling through optical positioning of a quantum dot in a photonic crystal cavity. Appl. Phys. Lett. 94, 111115 (2009).

[b18] Dousse A. *et al.* Controlled light-matter coupling for a single quantum dot embedded in a pillar microcavity using far-field optical lithography. Phys. Rev. Lett. 101, 267404 (2008). 19437672 10.1103/PhysRevLett.101.267404

[b19] Luxmoore I. J. *et al.* Optical control of the emission direction of a quantum dot. Appl. Phys. Lett. 103, 241102 (2013).

[b20] Gammon D. *et al.* Fine structure splitting in the optical spectra of single GaAs quantum dots. Phys. Rev. Lett. 76, 3005–3008 (1996). 10060846 10.1103/PhysRevLett.76.3005

[b21] Stepanov P. *et al.* Quantum dot spontaneous emission control in a ridge waveguide. Appl. Phys. Lett. 106, 041112 (2015).

[b22] Tighineanu P., Sørensen A. S., Stobbe S. & Lodahl P. Unraveling the mesoscopic character of quantum dots in nanophotonics. Phys. Rev. Lett. 114, 247401 (2015). 26197011 10.1103/PhysRevLett.114.247401

[b23] Faraon A. *et al.* Dipole induced transparency in waveguide coupled photonic crystal cavities. Opt. Express 16, 12154–12162 (2008). 18679491 10.1364/oe.16.012154

[b24] Grazioso F., Patton B. R. & Smith J. M. A high stability beam-scanning confocal optical microscope for low temperature operation. Rev. Sci. Instrum 81, 093705 (2010). 20886985 10.1063/1.3484140

[b25] Johnson S. S. & Joannopoulos J. J. Block-iterative frequency-domain methods for Maxwell's equations in a planewave basis. Opt. Express 8, 173–190 (2001). 19417802 10.1364/oe.8.000173

[b26] Lumerical FDTD Solutions. Lumerical Solutions, Inc. http://www.lumerical.com/tcad-products/fdtd/ (2014).

