# Supplementary material for: Chirality of nanophotonic waveguide with embedded quantum emitter for unidirectional spin transfer
Source: Nat Commun. 2016 Mar 31;7:11183. doi: 10.1038/ncomms11183 (PMC4821884; doi:10.1038/ncomms11183)
Supplement: Supplementary Information — Supplementary Figures 1-10, Supplementary Notes 1-4 and Supplementary References. [file ncomms11183-s1.pdf]

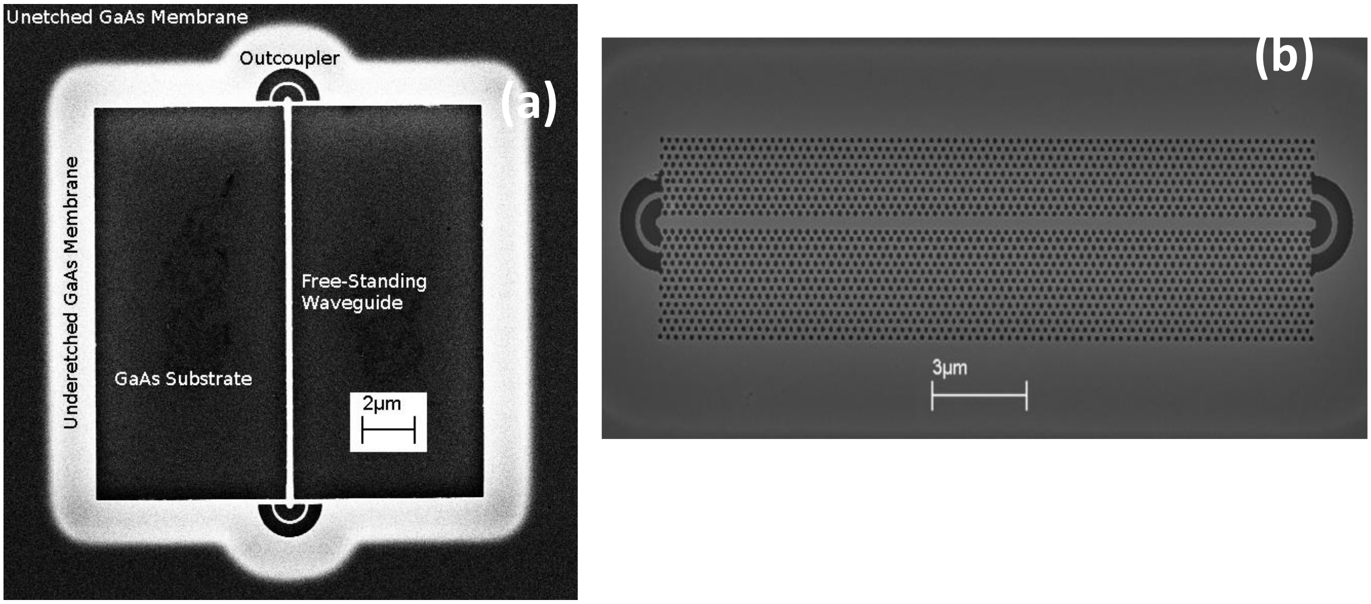

Supplementary Figure 1. Nanobeam and photonic crystal waveguide devices.

Examples of SEM images of (a) suspended nanobeam waveguide and (b) photonic crystal waveguide used in experiments.

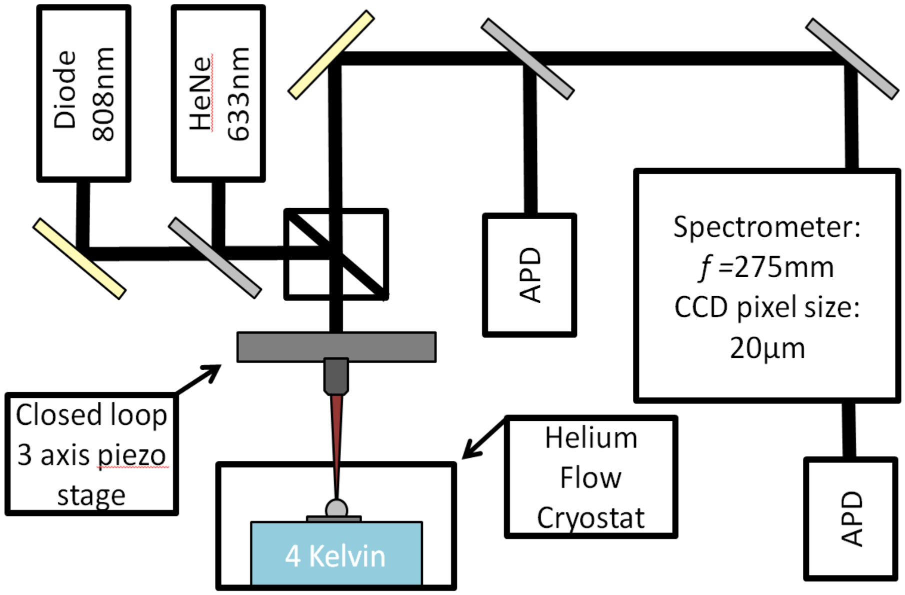

Supplementary Figure 2. **Quantum dot registration experimental setup.**

An 808 nm diode laser is used to excite the sample through a 50:50 cubic beam splitter. A 50x objective with NA of 0.5 is used to focus on to the sample. The collected light is split by a 92:8 pellicle beam splitter (transmission : reflection) and is detected via two independent APDs. The sample is mounted in an Oxford Instruments continuous flow cryostat and cooled to 4 K.

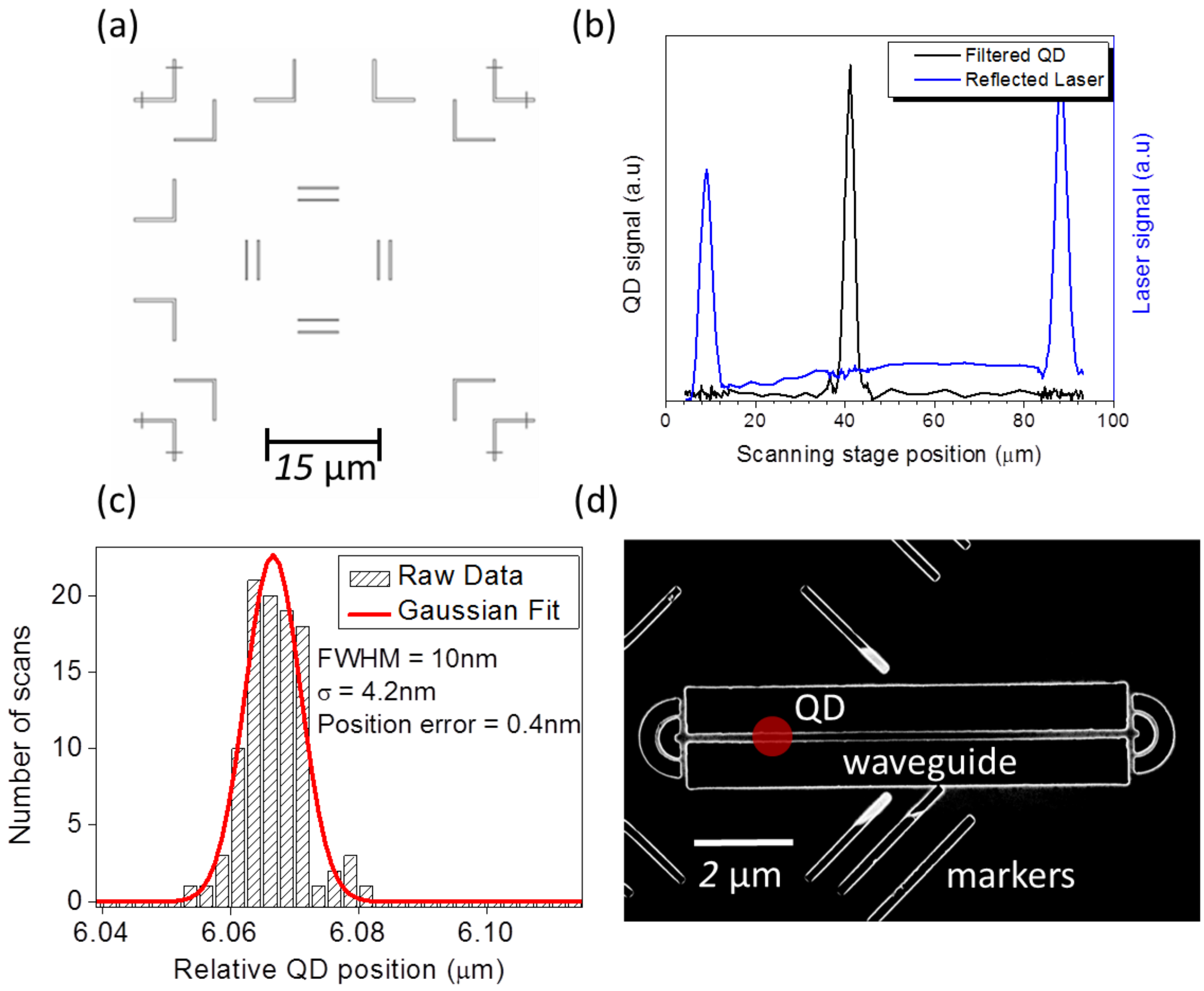

Supplementary Figure 3. Quantum dot registration accuracy.

(a) Schematic showing design of gold markers on the surface of the sample. (b) Example line scan showing how the vertical position is determined relative to a gold marker. The bright laser peaks are used to determine the magnification of the system due to the SIL (typically ~5.4). (c) Histogram of relative quantum dot positions measured for a single dot in the vertical axis. An error of 0.4 nm is calculated as $\sigma / \sqrt{N}$ where σ is the standard deviation and N is the total number of scans. (d) SEM image of the fabricated waveguide with QD position marked with a red circle.

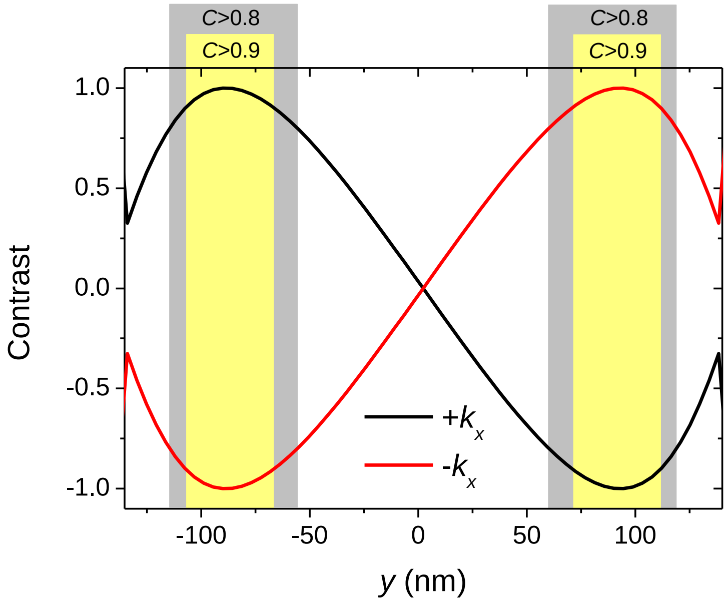

Supplementary Figure 4. Spin readout contrasts in nanobeam waveguide.

Calculated contrast for infinite length nanobeam waveguide $\mathbf{d}_+$ (σ^+ polarised) and $\mathbf{d}_-$ dipoles (σ^- polarised) for propagation to the left ($-k_x$) is shown in red and to the right ($+\,k_x$) is shown in black.

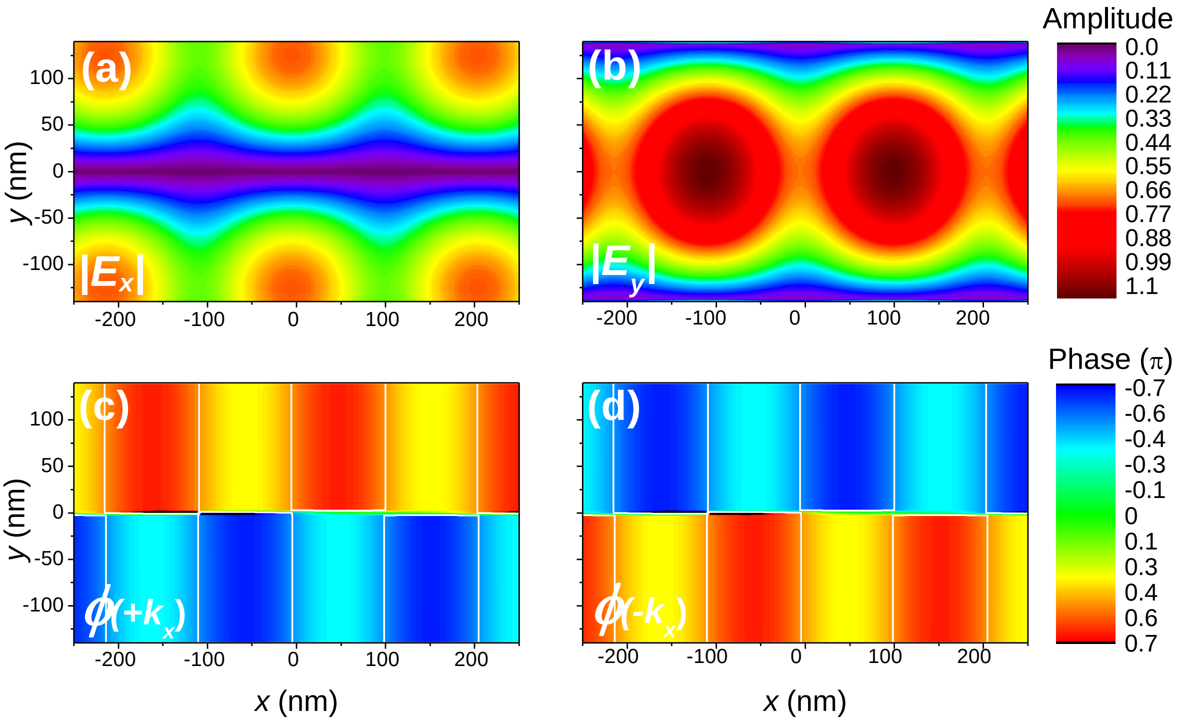

Supplementary Figure 5. Electric field distributions in nanobeam with out-couplers.

Calculated electric field profiles for (a) E_x and (b) E_y components for the nanobeam waveguide terminated by the out-couplers. Phase between the electric field components for the same waveguide for mode excitation from the (c) left and (d) right. White vertical contours indicate where local field phase $|\varphi(x)| = \pi/2$.

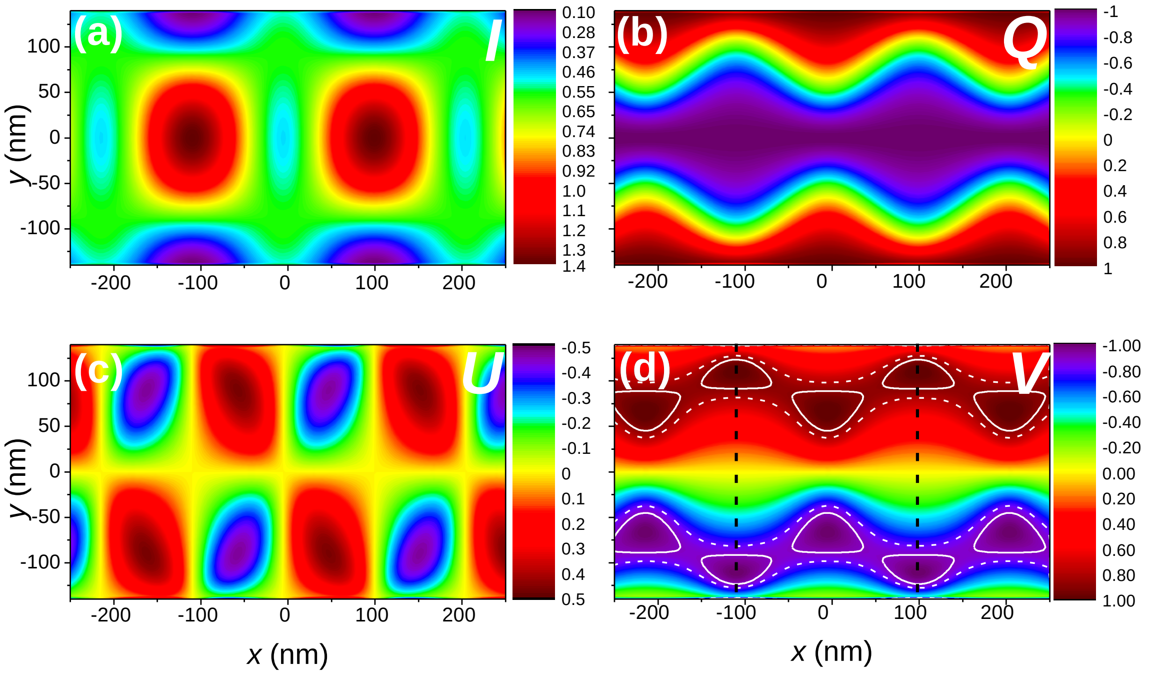

Supplementary Figure 6. Calculated Stokes parameters for the fields of the nanobeam waveguide terminated by out-couplers.

(a) Intensity, *I*. (b) Degree of linear polarisation, *Q*. (c) Degree of diagonal polarisation, *U*. (d) Degree of circular polarisation, *V*. Solid (dashed) white contours enclose regions where the degree of circular polarisation exceeds 90% (80%). Black dashed lines show the ‘unit cell’ used for statistical calculations in the main text.

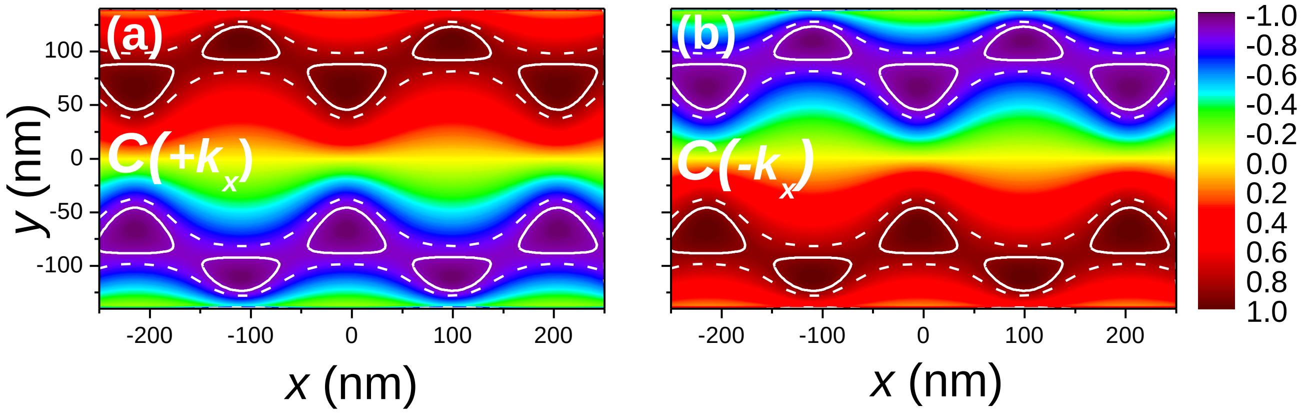

Supplementary Figure 7. Spin readout for the nanobeam waveguide terminated by out-couplers.

Calculated contrast of spin readout for the nanobeam waveguide terminated by out-couplers for propagation to the (a) right and (b) left. White solid (dashed) contours enclose regions of contrast exceeding 90% (80%).

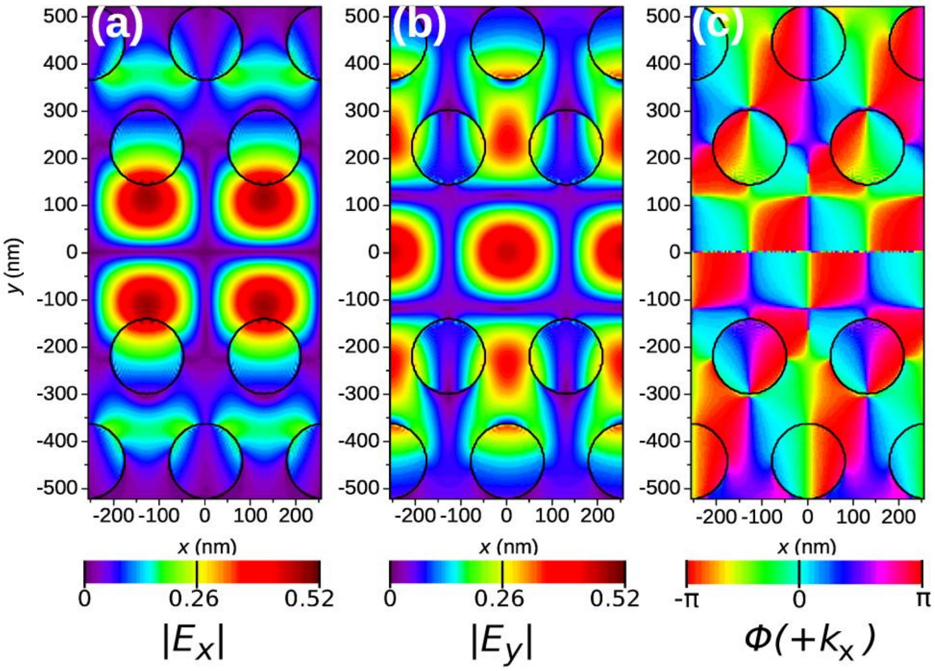

Supplementary Figure 8. Calculated electric field profiles for the infinite photonic crystal waveguide.

(a) E_x and (b) E_y components. Phase between the electric field components for the same waveguide for mode propagation to the right ($+k_x$). For propagation to the left ($-k_x$), the phase is inverted. Black contours enclose air holes of photonic crystal membrane.

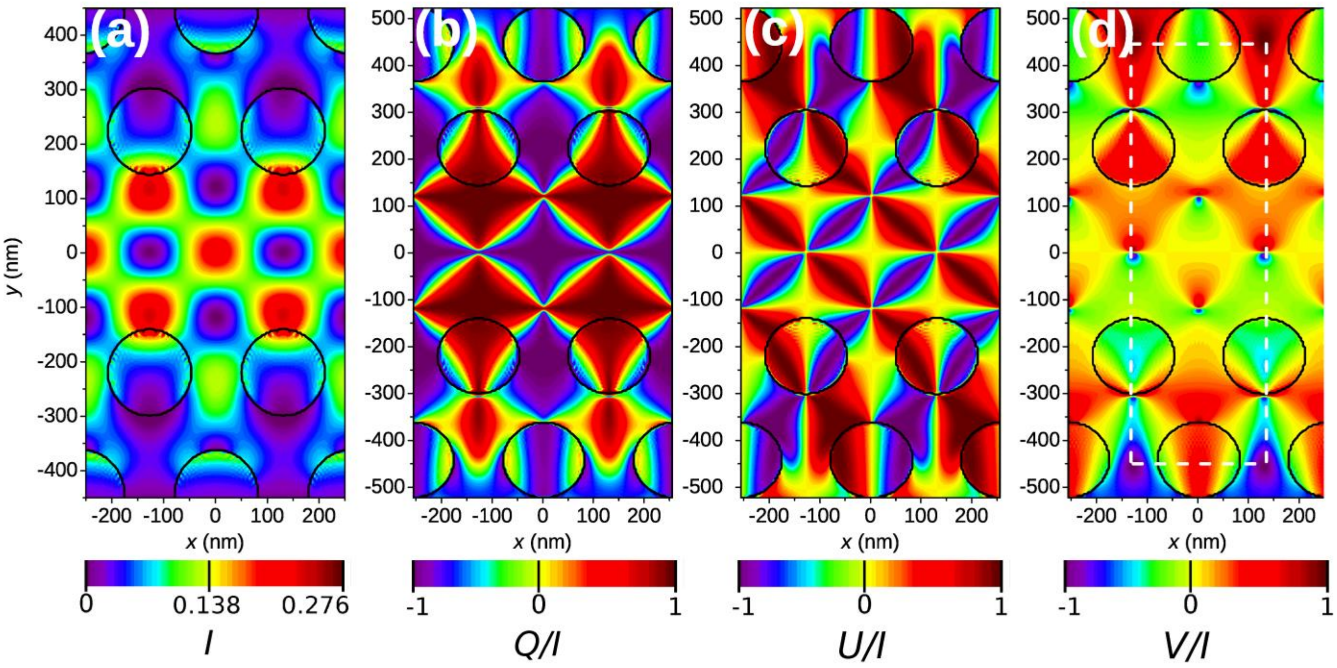

Supplementary Figure 9. Calculated Stokes parameters for the fields of the photonic crystal waveguide.

(a) Intensity, *I*. (b) Degree of linear polarisation, *Q*. (c) Degree of diagonal polarisation, *U*. (d) Degree of circular polarisation, *V*. Black contours enclose etched (air) regions of the photonic crystal lattice. White dash-dot region shows unit cell used for statistical calculations in the main text. Data in the air holes was omitted from calculations. Regions of high circular polarisation degree are not highlighted in (d) as these regions are of small area, but can been seen as points at the extremes of the colour scale.

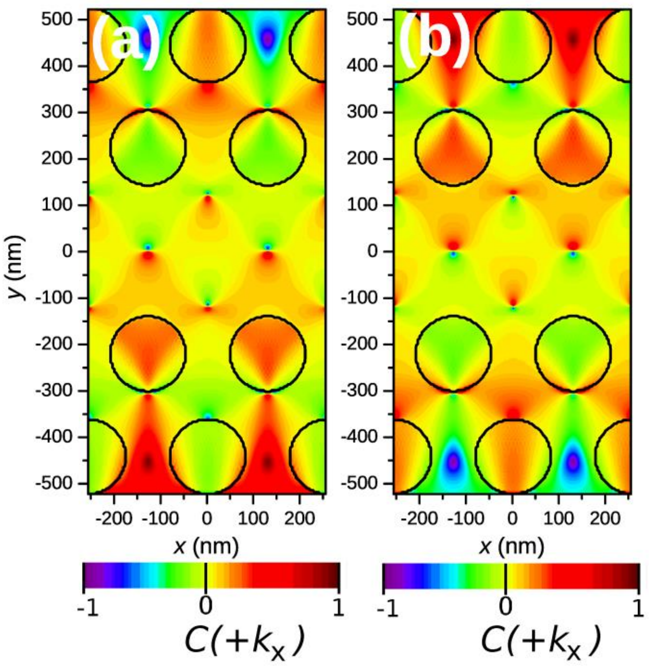

Supplementary Figure 10. Spin Readout in photonic crystal waveguide.

Calculated contrast of spin readout for the photonic crystal waveguide for propagation to the (a) right and (b) left.

Supplementary Note 1. Approximate solution for the field profiles of Suspended Nanobeam Waveguides

An approximate form of the fields of the suspended nanobeam waveguides can be found by making a few simplifying assumptions and solving Maxwell's equations with appropriate boundary conditions. Firstly, the fields are assumed to be of the form $\mathbf{E}(x, y, z, t) = \mathbf{E}(y, z)\, e^{i\omega t+ikx}$, where $k = 2\pi/\lambda$ is the propagation wavenumber of the guided mode with wavelength λ and angular frequency ω (see Fig. 2a of main text for schematic of waveguide and axes). The electric dipole of the quantum dot is constrained to the *xy*-plane so that the only modes of interest are those which have an even parity in *z* such that $\mathbf{E}(z) = \mathbf{E}(-z)$ [1]. The fields therefore have a cosine-like z-dependence, and at $z = 0$ (the centre of the GaAs membrane) $\mathrm{d}\mathbf{E}/\mathrm{d}z = \mathrm{d}\mathbf{H}/\mathrm{d}z = 0$. The fields in the $z = 0$ plane can thus be written as:

$$kE_y - i\frac{\mathrm{d}E_x}{\mathrm{d}y} = \mu\omega H_z \qquad (1)$$

$$\frac{\mathrm{d}H_z}{\mathrm{d}y} = i\omega E_x \qquad (2)$$

$$kH_z = -\varepsilon\omega E_y \qquad (3)$$

The fundamental TE-like mode of the suspended nanobeam waveguide has odd parity in *y* so $E_y(y) = E_y(-y)$ and $E_x(y) = -E_x(-y)$. This means that the fields internal to the waveguide are given by:

$$E_y(y, z = 0) \propto \cos(\tilde{k}y) \qquad (4)$$

$$E_x(y, z = 0) \propto -i\sin(\tilde{k}y) \qquad (5)$$

$$H_z(y, z = 0) \propto -\cos(\tilde{k}y) \qquad (6)$$

where $\tilde{k}=k\sqrt{(n^2+1)}$ and n=3.4 is the refractive index of GaAs. There are important conclusions to be drawn from these equations: i) the only fields in the QD plane are E_x, E_y and H_z; ii) there is a $|\varphi| = \pi/2$ phase shift between E_x and E_y as shown in Figure 2b of the main text; iii) the sign of φ is inverted when either the propagation direction ($\pm k$) or dipole position ($\pm y_d$) change sign. These properties are confirmed by numerical simulations of the suspended nanobeam in three dimensions as presented in the main text in Figure 2b and discussed in Supplementary Note 2 below.

Supplementary Note 2. Simulations of Infinite Suspended Nanobeam Waveguide.

Fully-vectorial eigenmodes of Maxwell's equations with periodic boundary conditions were computed by preconditioned conjugate-gradient minimization of the block Rayleigh quotient in a plane-wave basis, using a freely available software package [2]. The complex fields of the fundamental propagating mode of a 2D *yz*-cross section of the suspended nanobeam waveguide were calculated for λ = 930 nm using the dimensions given in Methods, the results of which are shown in Figure 2b of the main text. The degree of readout contrast for left and right propagation was calculated from the modal fields of Figure 2b using (7) and (8) respectively:

$$C_{\mathrm{L}} = \frac{(\Gamma_{\mathrm{L}}^{-} - \Gamma_{\mathrm{L}}^{+})}{(\Gamma_{\mathrm{L}}^{-} + \Gamma_{\mathrm{L}}^{+})}, \tag{7}$$

$$C_{\mathrm{R}} = \frac{(\Gamma_{\mathrm{R}}^{+} - \Gamma_{\mathrm{R}}^{-})}{(\Gamma_{\mathrm{R}}^{+} + \Gamma_{\mathrm{R}}^{-})}. \tag{8}$$

The QD emission rate [3], $\Gamma \propto \mathrm{Re}\{\mathbf{d}^{*}\cdot\mathbf{E}\}^{2}$, where the dipole moment is $\mathbf{d}_{\pm}=d(\mathbf{e}_{x} \pm i\, \mathbf{e}_{y})$ and the local electric fields are $\mathbf{E} = |E_x|\, \mathbf{e}_x + |E_y|\, e^{i\varphi}\, \mathbf{e}_y$. The dipole moment $\mathbf{d}_{+}$ ($\mathbf{d}_{-}$) denotes the dipole moment of a σ^{+} (σ^{-}) circularly polarised dipole source. The calculated contrast for both propagation directions is shown in Supplementary Figure 4. The fractional area over which contrast exceeding 90% (80%) is observed is 29% (41%) for the infinite nanobeam waveguide.

Supplementary Note 3. Simulations of Out-coupler Terminated Suspended Nanobeam Waveguide.

Presentations of simulations in the main text include data calculated for waveguides terminated by out-coupler gratings only in Fig. 3e. The out-coupler designs from [4] were used for the experiments presented in this paper so this section investigates the behaviour of a suspended nanobeam waveguide terminated by out-couplers of this design. Complete simulated data and analysis for the effects of the out-coupler gratings are considered here. The out-coupler gratings have back reflections. A commercial-grade simulator based on the finite-difference time-domain method was used to perform the calculations for the nanobeam waveguide terminated by grating out-couplers [5]. The waveguide dimensions were the same as specified in Methods. The waveguide mode was excited using a mode source and the field profiles recorded using a field monitor in the xy-plane (z = 0) cantered at x, y = 0. Due to the calculated reflectivity of ~15% from the out-coupler gratings, standing waves are formed in the waveguide. The calculated field profiles for the E_x and E_y field components internal to the waveguide are shown in Supplementary Figures 5(a) and 5(b) respectively for λ = 934 nm, corresponding to a Fabry-Perot resonance maximum. The calculated phase distributions for these fields are shown in Supplementary Figure 5(c) and 5(d) for excitation from the left and right sides of the waveguide respectively. Due to reflections from the out-couplers, a longitudinal phase variation is introduced where $0.345\pi \leq |\varphi(x)| \leq 0.655\pi$. Regions where the phase is locally $|\varphi(x)| = \pi/2$ are shown by vertical white contours in Supplementary Figure 5(c) and 5(d).

To gain a more intuitive insight into the effects of standing wave formation on the field polarization profile, Stokes parameters are calculated for the electric fields and displayed in Supplementary Figure 6. The Stokes parameters are defined in Eqs. (9)-(12) as:

$$I = |E_x|^2 + |E_y|^2 \tag{9}$$

$$Q = |E_x|^2 - |E_y|^2 \tag{10}$$

$$U = 2\,\mathrm{Re}\{E_x E_y^*\} \tag{11}$$

$$V = -2\,\mathrm{Im}\{E_x E_y^*\} \tag{12}$$

The intensity I is shown in Supplementary Figure 6(a), the degree of linear polarisation Q in (b), the degree of diagonal polarisation U in (c) and circular polarisation V in (d). Regions where the circular polarisation degree approaches unity are herein referred to as 'C-points'.

As with the infinite length nanobeam waveguide, close to the centre of the waveguide the fields are linearly polarised along y. The standing waves of the waveguide establish regions of diagonal polarisation for $|y|>0$ with periodicity along x. The regions of high ($|V|>0.9$) circular polarisation are also periodic along the waveguide axis. However for $|y|$ ~ 90 nm the circular polarisation degree remains above 0.87 for all values of x. At the same displacement, the infinite suspended nanobeam case yields a circular polarisation degree of >0.99 continuously along x. The modulation of the mode profiles from back reflections from the out-couplers acts to reduce the maximum degree of circular polarisation at a given position in the waveguide. Appropriately designed impedance matched out-couplers can eliminate these back reflections and restore behaviour closer to the infinite suspended nanobeam waveguide case.

Calculation of the contrast of spin readout as in the previous section yields spatial profiles which exhibit the same spatial dependence as the degree of circular polarisation of the suspended nanobeam

waveguide fields, as shown in Supplementary Figure 7. Despite the effects of the out-couplers, high fidelity (C > 0.99) spin readout contrast is observed along the waveguide as demonstrated experimentally in Figure 3d and in simulations presented in Figure 3e of the main text. The fractional area over which contrast exceeding 90% (80%) is observed is 14% (34%) for the nanobeam waveguide terminated by out-coupler gratings. Compared to the infinite waveguide case these areas show a reduction in size of ~ 52% for regions exceeding 90% contrast (~ 17% for regions exceeding 80%) contrast due to back reflections from the out-couplers.

Supplementary Note 4. Simulations of Photonic Crystal Waveguide.

Fully-vectorial eigenmodes of Maxwell's equations with periodic boundary conditions were computed for the photonic crystal waveguide using a freely available software package [2]. The waveguide dimensions were the same as specified in Methods. The waveguide mode was excited using a mode source and the field profiles recorded using a field monitor in the *xy*-plane (z = 0) centred at *x*, *y* = 0. The Bloch periodicity of the photonic crystal leads to the formation of longitudinal and lateral variations of the field profiles. All figures presented for the photonic crystal are for infinite waveguides. The field profiles of the W1 photonic crystal waveguide mode are shown in Supplementary Figure 8 (a) and 8 (b) with the phase between them for propagation to the right ($+k_x$) in Supplementary Figure 8 (c).

The Stokes parameters are calculated for the electric fields of the photonic crystal waveguide mode at λ = 934 nm. The intensity is shown in Supplementary Figure 9(a), the degree of linear polarisation *Q* in (b), the degree of diagonal polarisation *U* in (c) and circular polarisation *V* in (d). The strong modulation of the modal fields by the photonic crystal lattice produces regions of fully diagonally polarised fields. There are 6 C-points (C > 90%) per unit cell for the photonic crystal waveguide compared to the quasi-continuous bands of high circular polarisation for the suspended nanobeam waveguide with out-couplers. In addition, for the W1 photonic crystal waveguide, these points occur near to regions of low field intensity so a low dipole coupling efficiency is expected.

The spin readout contrast for circularly polarised dipole sources was calculated for the photonic crystal waveguide fields, using the same method as for the suspended nanobeam in the previous section. The results of these calculations are plotted in Supplementary Figure 10. The calculated spatial distribution of the contrast exhibits the same spatial profile as the degree of circular polarisation *V* from Supplementary Figure 9(d). This is consistent with experimental results in Figure 4d which demonstrates no QDs with a contrast of spin readout exceeding 0.7. The reduced average fidelity for the photonic crystal waveguide relative to the suspended nanobeam waveguide is a direct consequence of the limited number and size of the chiral points provided by the modal fields of the photonic crystal waveguide. The fractional area over which contrast exceeding 90% (80%) is observed is 0.8% (1.5%) for the photonic crystal waveguide, which is around 5% of the area observed in the suspended nanobeam waveguide with out-couplers.

Supplementary References

1 Makhonin, M. N., Dixon, J. E., Coles, R. J., Royall, B., Luxmoore, I. J., Clarke, E., Hugues, M., Skolnick, M. S. & Fox, A. M. Waveguide coupled resonance fluorescence from on-chip quantum emitter, *Nano Lett.* **14**, 6997-7002 (2014)

2 Johnson, S. S. & Joannopoulos, J. J. Block-iterative frequency-domain methods for Maxwell's equations in a planewave basis, *Opt. Express* **8**, 363–376 (2001)

3 Novotny, L. & Hecht, B. Principles of Nano-Optics. Cambridge University Press. (2006)

4 Faraon, A., Fushman, I., Englund, D., Stoltz, N., Petroff, P.& Vuckovic, J. Dipole induced transparency in waveguide coupled photonic crystal cavities, *Opt. Express* **16**, 12154–12162 (2008)

5 Lumerical FDTD Solutions. Lumerical Solutions, Inc. http://www.lumerical.com/tcad-products/fdtd/ (2014)
